# Serum aspirin esterase is strongly associated with glucose and lipids in healthy subjects: different association patterns in subjects with type 2 diabetes mellitus

**DOI:** 10.1186/1758-5996-2-50

**Published:** 2010-07-27

**Authors:** Kazuhiko Kotani, Satoshi Kimura, Tetsu Ebara, Russell Caccavello, Alejandro Gugliucci

**Affiliations:** 1 Department of Clinical Laboratory Medicine, Jichi Medical University, Tochigi, Japan; 2 Glycation, Oxidation and Disease Laboratory, Touro University-California, CA, USA; 3 Department of Laboratory Medicine and Central Clinical Laboratory, Showa University Northern Yokohama Hospital, Kanagawa, Japan; 4 Department of Internal Medicine, Showa University Northern Yokohama Hospital, Kanagawa, Japan

## Abstract

**Background:**

Aspirin esterase (AE) activity can account for part of aspirin pharmacokinetics in the circulation, possibly being associated with the impairment of aspirin effectiveness as an inhibitor of platelet aggregation.

**Aims:**

The study was aimed at investigating the correlations of serum AE activity with cholinesterase (ChE) and metabolic variables in healthy subjects in comparison to subjects with type 2 diabetes mellitus (T2DM).

**Methods:**

In cardiovascular disease-free T2DM subjects and healthy controls, the AE activity levels and/or the correlation patterns between AE and the other variables were analyzed.

**Results:**

Neither AE nor ChE activities were higher in the subjects with T2DM. Serum AE activity strongly correlated with ChE as well as glucose/lipids variables such as total cholesterol and triglyceride in healthy subjects, while the correlations between AE and glucose/lipids variables were not present in T2DM subjects.

**Conclusions:**

These data may reflect the pathophysiological changes between healthy and T2DM subjects. Our data may thus provide the basis for future studies to unravel the mechanisms.

## Background

Aspirin (acetylsalicylic acid), by virtue of its antipyretic, analgesic, anti-inflammatory and anti-platelet actions, is one of the most widely employed drugs in the prevention of cardiovascular disease. While its prophylactic use against athero-thrombosis is well-documented, the optimal dose that confers maximal anti-platelet action without increased risk of bleeding remains to be determined [1,2]. Serum aspirin esterase (AE) activity may account for part of aspirin pharmacokinetics and has been proposed as one source of variation in aspirin effectiveness [3].

Type 2 diabetes mellitus (T2DM) is a common health problem associated with cardiovascular disease, with an increasing incidence worldwide [4]. One study has recently reported an increased level of AE activity in patients with T2DM, and it has been suggested that this may be modified by lipoprotein-cholesterol metabolism in these patients [5]. In addition, serum cholinesterase (ChE) has shown a tight correlation with AE and an association with the dyslipoproteinemia of the metabolic syndrome [5,6]. However, these relationships remain yet to be fully established. We thus conducted this study to shed further light on the correlation of AE with ChE and metabolic variables, including lipids, in healthy subjects in comparison to subjects with T2DM.

## Methods

In this case-control observational study, twenty-seven subjects with T2DM, who were on stable metabolic control and treated with hypoglycemic agents, were enrolled from our outpatient population consulting the Showa University Northern Yokohama Hospital, Japan. Twenty control subjects in the study were recruited from an asymptomatically healthy population with no history of diabetes mellitus. All subjects were free of acute illness, and those with a history of cardiovascular disease and malnutrition as well as with a prescription of aspirin were excluded. The study was approved by the institutional review board of Showa University and each subject gave informed consent.

A fasting blood sample was obtained by venipuncture and dry tubes were used for serum or for plasma sodium citrate (5 mmol/L final concentration) or sodium EDTA (5 mmol/L final concentration) were added as anti-coagulants. Blood was centrifuged at 800 × g at 4°C for 15-minutes, and separated serum or plasma was immediately analyzed. Serum lipid panels such as total cholesterol, high-density lipoprotein (HDL) cholesterol and triglyceride, as well as serum albumin and plasma glucose, were enzymatically measured. Hemoglobin A1c (HbA1c) were measured by an HPLC method. Serum AE activity was measured kinetically by a modification of the procedure described by Sorensen [7]. The reagent buffer contains Tris-HCl (0.6 mol/L) and CaCl_2 _(0.4 mol/L) in pH 7.6-7.7. Substrate is 555 mmol/L acetylsalicylic acid (1 g in 10 mL 100% ethanol for the stock solution). The method was modified in our laboratory toward a more precise kinetic method adapted to 96-well microplates. Briefly, 15 μL sample (serum) were pipetted per well (in duplicate) into a 96-well UV flat bottom plate (Thermo-8404). Stock substrate solution was diluted 1 in 50 into reagent buffer, and then 200 μL of this final substrate solution was added per well using a multi-channel pipette. The runs were blanked against reagent (to control for spontaneous hydrolysis of acetylsalicylic acid). Plates were placed in a temperature-controlled plate reader SpectraMax UV with SOFTmax PRO software (Molecular Devices, Sunnyvale, CA, USA). Samples were kinetically read at 300 nm at 37°C for 15-minutes. Results are expressed in nmol of acetylsalicylic acid hydrolyzed per minute and per milliliter (nmol/mL/min). The intra-assay CV (coefficient of variation) is 4%, and the inter-assay CV is 5%. Additionally, serum ChE (butyrylcholinesterase) activity was determined using the standard procedure as previously described [8]. The assay uses the thiol ester butyrilcholine. ChE hydrolyses the ester to produce thiocholine and acetate. The thiocholine in turn reduces the dithiobis-nitrobenzoic acid (DTNB) liberating nitrobenzoate, which absorbs at 405 nm. Briefly, 5 μL sample (serum) were pipetted per well (in duplicate) into a 96-well plate. The buffer (100 mmol/L NaHPO_4 _in pH 7.0-8.0), substrate (7.5 mmol/L butyrylthiocholine iodide in H_2_O) and DTNB (10 mmol/L 5, 5'-dithiobis-2-nitrobenzoic acid (3.96 mg/mL) in 100 mmol/L buffer containing 1.5 mg NaH_2_CO_4_) were mixed to give a final concentration of 0.1 mmol/L butyrylthiocholine. The runs were blanked against reagent, and plates were placed in a temperature controlled plate reader SpectraMax UV. Samples were kinetically read at 405 nm at 37°C for 3-minutes. Results are expressed in U/L. The intra-assay and inter-assay CVs are 0.8 and 6.3%, respectively.

Data were expressed as mean ± standard deviation or median (interquartile range). For the between-group differences, unpaired t-test or χ^2^-test was used. For the correlations between AE and the other variables, we used single and multiple linear regression models. Considering the basic confounders and the co-linearity between the measured variables (in particular, glucose/lipids and ChE with each other: e.g., ChE *vs*. total cholesterol, r = 0.57; triglyceride, r = 0.76; fasting glucose, r = 0.45), the two adjusted-linear regression models (age- and gender-adjusted model, as well as age-, gender- and body mass index-adjusted model) were set out. Because triglyceride and AE had a skewed distribution, the values were logarithm-transformed in analyzing. A p value < 0.05 was considered significant.

## Results

The subject characteristics are listed in Table 1. Although the group of T2DM had significantly higher levels of fasting glucose and HbA1c as well as significantly lower levels of HDL cholesterol than the control group, AE and ChE levels did not significantly differ between the two groups. As shown in Table 2, in single correlation tests (Model 1), in the control group, AE was significantly and positively correlated with fasting glucose, total cholesterol and triglyceride, respectively. While AE was inversely correlated with HDL cholesterol, this correlation did not reach a statistical significance level. In contrast, in the group of T2DM, there were no significant correlations between these variables and AE. A significant positive correlation between AE and ChE was observed in both groups of T2DM and control, while the correlation level appeared somewhat high in the control group. The respective correlation patterns in the group of T2DM and control remained to be similarly continued, even after adjusting for age and gender (Model 2), as well as age, gender and body mass index (Model 3).

**Table 1:** Clinical characteristics of type 2 diabetic subjects and control subjects

Variables	Controls (N = 20)	Type 2 Diabetics (N = 27)	p value
Age, years	60.1 ± 14.3	55.9 ± 10.0	0.24
Men/women, n	8/12	18/9	0.07
Body mass index, kg/m^2^	22.1 ± 3.7	22.8 ± 2.6	0.46
Fasting glucose, mmol/L	5.41 ± 0.79	9.13 ± 3.66	< 0.0001**
HbA1c, %	5.0 ± 0.4	7.6 ± 1.3	< 0.0001**
Total cholesterol, mmol/L	5.54 ± 1.10	5.28 ± 0.96	0.48
Triglyceride, mmol/L	1.04 (0.84-1.45)	2.11 (1.93-2.34)	0.13
HDL cholesterol, mmol/L	1.76 ± 0.41	1.40 ± 0.38	0.006**
Albumin, μmol/L	629 ± 27	605 ± 44	0.07
ChE, U/L	319 ± 73	360 ± 74	0.07
Aspirin esterase (nmol/mL/min)	39.2 (32.5-48.7)	41.6 (37.1-46.5)	0.66

HbA1c: hemoglobin A1c, HDL: high-density lipoprotein, ChE: cholinesterase. Data are presented as mean ± standard deviation, median (interquartile range) or number. Triglyceride and aspirin esterase were log-transformed because of their skewed distribution in the statistical analyses. Significance level (t-test or χ^2^-test between the group with controls and type 2 diabetes): ** p < 0.01.

**Table 2:** Correlation patterns for aspirin esterase to the other variables in type 2 diabetic subjects and in control subjects

	Controls			Type 2 diabetics		
		
Variables	Model 1	Model 2	Model 3	Model 1	Model 2	Model 3
Age, years	-0.11(0.64)	-	-	0.01(0.96)	-	-
Gender, men	0.10(0.69)	-	-	-0.07(0.74)	-	-
Body mass index, kg/m^2^	0.08(0.76)	0.07(0.80)	-	0.28(0.32)	0.42(0.25)	-
Fasting glucose, mmol/L	0.54(0.014)*	0.65(0.006)**	0.65(0.008)**	0.16(0.44)	0.15(0.48)	-0.04(0.85)
HbA1c, %	-0.18(0.45)	-0.16(0.61)	-0.14(0.66)	0.09(0.67)	0.07(0.75)	0.03(0.93)
Total cholesterol, mmol/L	0.58(0.007)**	0.67(0.004)**	0.69(0.006)**	-0.06(0.76)	-0.07(0.74)	-0.21(0.56)
Triglyceride, mmol/L	0.85(< 0.0001)**	0.85(< 0.0001)**	0.86(< 0.0001)**	-0.07(0.72)	-0.06(0.77)	-0.16(0.61)
HDL cholesterol, mmol/L	-0.43(0.09)	-0.50(0.12)	-0.56(0.13)	-0.04(0.86)	-0.25(0.29)	-0.08(0.86)
Albumin, μmol/L	0.44(0.06)	0.43(0.07)	0.43(0.10)	0.03(0.90)	0.09(0.71)	-0.03(0.94)
ChE, U/L	0.78(< 0.0001)**	080(< 0.0001)**	0.80(0.001)**	0.70(< 0.0001)**	0.70(< 0.0001)**	0.75(0.02)*

HbA1c: hemoglobin A1c, HDL: high-density lipoprotein, ChE: cholinesterase. Data are presented as the single linear regression coefficients (p value) in Model 1, the age- and gender-adjusted linear regression coefficients (p value) in Model 2 and the age-, gender- and body mass index-adjusted linear regression coefficients (p value) in Model 3, respectively. Triglyceride and aspirin esterase were log-transformed because of their skewed distribution in the single and multiple linear regression analyses. Significance level: * p < 0.05, ** p < 0.01.

## Discussion

We found that serum AE activity did not differ between T2DM subjects and healthy subjects. This is in contrast with a previous report showing an increased AE activity in patients with T2DM [5]. The inconsistency may partly stem from the differences in studied populations including ethnicity [9] and/or their respective degree of metabolic control. Our studied population seemed to be in good metabolic control as shown by their body mass index, HbA1c and lipids levels. Since T2DM is a syndrome with a wide spectrum of metabolic disturbances and etiologies [10,11], our results can not completely rule out the earlier hypothesis that the patients with T2DM are aspirin-resistant due to increased AE activity, while our data do not support them. Further controlled studies in various populations are needed to solve this controversy. Second, we also showed that serum ChE levels did not obviously differ between the subjects with T2DM and healthy subjects, but they had a very strong correlation with AE activity. This may be in line with the previous work [7]. It must be noted that, as described in the Methods section, the two enzyme activities are measured with different substrates and detection methods, therefore the correlation is not due to an analytical bias. One of the possible mechanisms to explain the correlation is the fact that aspirin is hydrolyzed in the circulation by two distinct pathways of a spontaneous pH-dependent hydrolysis as well as an enzymatic hydrolysis by plasma and erythrocyte esterases, and that the plasma system consists, in part, of butyrylcholinesterase as well as albumin [12].

Thirdly, more interestingly, when we correlated AE activity in healthy subjects with the other variables and even when controlling for confounders, we found a significant close correlation of fasting glucose, total cholesterol and triglyceride with AE activity, respectively. This is the first new finding in our work. In contrast to the correlation between glucose and AE, the reason for a non-significant correlation between HbA1c and AE remains unclear. This may be related to the fact that fasting plasma glucose is a one-point measure while HbA1c is reflective of mean glucose condition over a period of 2-3 months. Possibly, our data imply that short-term changes in glucose, which may be regulated by insulin and/or glucagon changes, modify AE activity, while adaptations resulting from long-term adjustments can act differently for both analytes.

Moreover, surprisingly, the strong association between AE and glucose/lipids variables we observed in our control population was not present in subjects with T2DM. The present data suggest that the metabolic changes specifically found in T2DM (i.e., higher glycemic excursions, lower HDL cholesterol concentrations) [10,11] may cancel the link between glucose/lipids and AE activity. This second new finding of our work is puzzling and therefore interesting; the mechanisms remain to be explored.

This study had potential limitations such as a small sample-size design. However, given the clear and significant findings, this may lay the ground for further studies on the pathophysiology of the link between AE and metabolic variables.

## Conclusions

In summary, the present study shows that serum AE activity strongly correlates with ChE as well as glucose/lipids variables in healthy subjects. Neither AE nor ChE activities increase in the subjects with T2DM, and the correlations between AE and glucose/lipids variables are not present in these subjects. These data may open the field for future studies to unravel the mechanisms.

## Competing interests

The authors declare that they have no competing interests.

## Authors' contributions

All authors contributed the intellectual development of this work, and approved the final manuscript. SK and TE collected the samples. KK, SK, RC and AG analyzed the samples and data. KK, SK and AG wrote the manuscript. KK, RC and AG searched the literature, and ET and RC reviewed the manuscript.
